# HIV cell-to-cell spread and innate immune responses

**DOI:** 10.1186/1742-4690-10-S1-O34

**Published:** 2013-09-19

**Authors:** Olivier Schwartz

**Affiliations:** 1 Institut Pasteur, Department of Virology, Virus & Immunity Unit, Paris, 75015, France

## 

Our research is aimed at understanding the interplay between viruses and the immune system. HIV cell-to-cell transmission is a major mechanism of viral spread. We are studying how antiviral molecules, such as broadly neutralizing antibodies and restriction factors, known to inhibit infection with cell-free virions, interfere with HIV-1 cell-to-cell transmission. Moreover, we previously showed that HIV-1 infected lymphocytes are more potent inducers of type-l IFN than free virions. There are target cell-type differences in the recognition of infected lymphocytes. In primary pDCs and pDC-like cells, recognition occurs in large part through TLR7. In myeloid DCs, and other cells that lack TLR7, recognition is independent of TLR7, and occurs in a large part through a cytoplasmic pathway that we are currently analyzing. Finally, we are examining how HIV-2 spreads from cell-to-cell and triggers type-l IFN in various primary cell types. Characterization of the mechanisms of innate recognition of HIV-infected cells allows a better understanding of the pathogenic and exacerbated immunologic events associated with HIV infection.

